# Cytometric Characterization of Main Immunocompetent Cells in Patients with Systemic Sclerosis: Relationship with Disease Activity and Type of Immunosuppressive Treatment

**DOI:** 10.3390/jcm8050625

**Published:** 2019-05-08

**Authors:** Olga Gumkowska-Sroka, Krystyna Jagoda, Aleksander Owczarek, Grzegorz Helbig, Joanna Giemza-Stokłosa, Przemysław J. Kotyla

**Affiliations:** 1 Department of Rheumatology, Voivodeship Hospital No. 5, 41-200 Sosnowiec, Poland; oag@op.pl; 2 Department of Hematology and Bone Marrow Transplantation, Medical Faculty in Katowice, Medical University of Silesia, 40-032 Katowice, Poland; ka-ja@wp.pl (K.J.); ghelbig@tlen.pl (G.H.); 3 Department of Statistics, Department of Instrumental Analysis, School of Pharmacy with the Division of Laboratory Medicine in Sosnowiec, Medical University of Silesia, 41-200 Sosnowiec, Poland; aowczarek@sum.edu.pl; 4 Department of Neurology, Regional Hospital in Oświęcim, 32-600 Oświęcim, Poland; giemza@mp.pl; 5 Department of Internal Medicine, Rheumatology and Clinical Immunology, Faculty in Katowice, Medical University of Silesia, 40-635 Katowice, Poland

**Keywords:** systemic sclerosis, flow cytometry, immunocompetent cells, disease activity, immunosuppression

## Abstract

Systemic sclerosis (SSc) is a connective tissue disease that is characterized by widespread skin and internal organ fibrosis vasculopathy and immune response abnormalities, including T, B, natural killer (NK), and natural killer T (NKT) cell involvement. The aim of the study was to investigate the immune cell profile in patients with systemic sclerosis in relation to the disease activity, severity, and antibody presence and their relation to the type of immunosuppressive treatment. Cytometric examination identified following cell lines: B cells (Breg, B memory, B mature) and plasmablasts, T cell, T double positive—Tdp, T double negative—Tdn, NK, and NKT cell and monocytes. The disease severity and activity were assessed based on the Medsger and the EULAR Scleroderma Trials and Research Group (EUSTAR) 2017 scales respectively. In the study, SSc patients were characterized by higher total lymphocyte count parallel to increased frequency of Ts and Th cells. In SSc patients, increment of Tdp and reduction of Tdn as well as NK and NKT cells were observed. Additionally in SSc patients the reduction of B memory was noted. Head to head comparison between cyclophosphamide (CYC) and mycophenolate mofetil (MMF) treatment showed a reduction of CD19^+^ cells, but increment of plasmablasts in CYC treated patients.

## 1. Introduction

Systemic sclerosis (SSc) is a connective tissue disease that is characterized by widespread skin and internal organ fibrosis, vasculopathy, and immune response abnormalities [1]. Although fibrosis is the most recognizable feature of the disease, internal organ involvement and subsequent damage is the leading cause of premature death in this population of patients. The pathogenesis of disease, although intensively studied, is not yet fully elucidated. At the current level of knowledge, it is commonly accepted that SSc belongs to the autoimmune-mediated disorders and generation of autoantibodies is an almost universal feature among patients with SSc [2]. Immunological activity, with T and B cell involvement, is considered to be a key component that ultimately leads to the vascular abnormalities and fibrosis observed in SSc. Studies performed thus far on T and B cell populations have given contradictory results. Moreover, most of the studies focused on early SSc patient or patients without any treatment. Less is known about T and B cells compartments in real world patients, who require these treatments. 

The role of B lymphocytes which may enhance the fibrosis has been intensively studied recently, and several mechanisms at least partially responsible for this process have been identified that include cytokine synthesis, intracellular interactions, participation in the regulation of the immune response and synthesis of autoantibodies [3]. With SSc, data on absolute and relative numbers of circulating B cell are contradictory, with some studies reporting similar, higher, or reduced numbers of B cells as compared to healthy counterparts [4,5,6]. The subtype of the disease (dcSSc vs. lcSSc) seems to have the lesser impact on B cell count. Contrary to this, there is a direct link between reduction in B cell counts and higher Rodnan score, pulmonary hypertension, and systemic sclerosis associated interstitial lung disease [3].

Regulatory B lymphocytes (Breg) play a key role in the maintenance of the immunological tolerance. This particular subset of B cells is able to suppress inflammatory response via IL-10 synthesis, which exerts potent anti-inflammatory activity. Reduction of Bregs has been reported in some studies in systemic sclerosis patients, especially in established disease and in SSc-interstitial lung disease [7]. Moreover circulating Bregs negatively correlate with C-reactive protein levels, and presence of SSc specific autoantibodies [7]. 

Immunological activation, especially of T cells, is considered a key point in the development of the vascular abnormalities and fibrosis. There is evidence that Th1 and Th2 cells contribute to the induction of pro-inflammatory (Th1) and pro-fibrotic (Th2) responses [8]. There is no agreement on T cell subpopulation counts in SSc. Some studies reported decreased levels of CD8^+^ but these findings were not confirmed by all [4,9]. Many controversies exist regarding the CD4^+^ T cell population in SSc patients. Higher frequencies and absolute counts of CD4^+^CD25^+^ and Treg were reported in the literature suggesting involvement of this arm of the immune response in progression of disease [10].

Most autoimmune diseases are associated with an increase in CD8^+^CD28^−^ T cells. Quantitative changes in this population have been observed in multiple autoimmune disorders including multiple sclerosis, Graves’ disease and rheumatoid arthritis. Some studies suggested that lymphocytes of a CD8^+^CD28^−^ phenotype may show regulatory properties. CD8^+^CD28^−^ T cells could represent a pathogenic T-cell subset in SSc, especially in the early stage of the disease, being increased in blood and skin SSc patients and correlating with disease duration and skin fibrosis extent. So far, few studies addressing the size of the CD8^+^CD28^−^ subpopulation in patients with SSc have been published, but the role of this T-cell subset in SSc is still unclear [11].

Only a few studies addressed the natural killer (NK) cell changes in SSc, again with contradictory results. Some studies suggested that NK cell changes may be relevant to the subtype of disease, being reported as reduced in dsSSc and normal in lsSSc. Those findings, however, were not reported in all studies [12].

The role of monocytes/macrophages in the pathogenesis of SSc is intensely debated. Taking into account the fibrotic processes that are the hallmark of the diseases, the profibrotic potential of monocytes has been studied. Quite recently, it was demonstrated that SSc patients had higher counts of monocytes in peripheral blood. Moreover two main disease complications, namely pulmonary arterial hypertension (PAH) and interstitial lung disease (ILD) were associated with increased number of CD16^+^ monocytes [13]. This finding supports the role of the innate immune response in driving the fibrotic process and endothelial dysfunction-related complications (PAH).

In this study, we focused on T and B cell abnormalities in an unselected SSc population at various stages of the disease and with internal organ involvement, who require standard therapy. The study was also aimed to detect a specific immune cell pattern that may characterize patients at various levels of disease activity, damage, and internal organ involvement. We also took a unique opportunity to study to what extent immunosuppressive treatments contribute to the restoration of normal immune cells pattern.

## 2. Materials and Methods

### 2.1. Patients

Forty-six adult patients with systemic sclerosis were recruited from the observational systemic sclerosis cohort at the Department of Internal Medicine Rheumatology and Clinical Immunology Medical University of Silesia, Katowice, Poland and screened for eligibility for the study. The diagnosis of disease was established according to American College of Rheumatology and European League against Rheumatism ACR/EULAR 2013 criteria [14]. The subtype of the disease was further characterized on the base of LeRoy criteria and patients with a diffuse and limited form of the disease were identified [15].

Patients were excluded from the study if they had an overlap syndrome (SSc with rheumatoid arthritis or SSc with systemic lupus erythematosus (SLE)), had received a course of B cell depletion therapy (anti-CD20), underwent bone marrow or stem cell transplantation, or suffered from any malignancies. We also excluded from the study pregnant and lactating women as well as subjects with limited venous access. Comprehensive medical history (including the time of onset of Raynaud symptoms and time of formal SSc diagnosis, previous and concomitant treatment), demographic data were taken in all patients, and detailed physical examination was done according to local guidelines. Routine laboratory tests including C3 and C4 complement levels, antinuclear antibody profile (antinuclear and anti-centromere antibody on Hep2 cells, anti Scl-70, Ro, La, and Ro52 by enzyme linked immunoabsorbent assay, ELISA) were done at the entry of the study.

All patients underwent routine echocardiographic examination with assessment of ejection fraction and of pulmonary arterial pressure. The pulmonary artery systolic pressure was estimated from the maximal continuous-wave Doppler velocity of the tricuspid regurgitation (TR) jet plus estimated right atrial pressure with the size of inferior vena cava and degree of change in caval diameter during respiration. TR V max values were averaged over more than 3 beats and values of more than 3.0 m/s (pulmonary arterial systolic pressure more than 41 mmHg) were regarded as possible PAH.

As a part of routine systemic sclerosis assessment, functional lung testing (FVC, TLC, and DLCO) was performed according to the American Thoracic Society/European Respiratory Society guidelines [8], using standard equipment. FVC was measured using a water-sealed spirometer, and the diffusing capacity for carbon monoxide (DLCO) value was obtained by the single-breath method and corrected for hemoglobin. The results were expressed as percentages of predicted values.

In or cohort of patients we usually perform chest high-resolution CT scans (HRCT) at the time of diagnosis and repeat every 12–18 months or earlier when necessary. All patients diagnosed with lung fibrosis had HRCT scans performed within period of 4 weeks prior to involvement to the study.

Clinical manifestations of SSc included Raynaud’s phenomenon, skin damage including pitting scars and active ulcers, and interstitial lung disease (defined as ground-glass opacities, sub-pleural reticulation with or without pleural irregularities, pleural traction, bronchiectasis, and/or honey combing on routine high-resolution CT scans). Esophageal manifestation was defined as either persistent esophageal dysphagia and/or as dilation of the esophagus on barium X-rays. 

Joint involvement was defined clinically, if patients suffered from arthralgia. Renal involvement was defined by either increased serum creatinine and/or by proteinuria of above 150 mg/day.

Patients underwent variable treatment regimens including administration of immunosuppressants where appropriate. Three months of stable current treatment was necessary for inclusion. Patients were permitted to take steroids, at a dose not exceeding 10 mg/day and immunosuppressants at standard doses (for methotrexate < 25 mg/week; mycophenolate mofetil < 2.0 g/day; azathioprine < 200 mg/day). Intravenous cyclophosphamide was also allowed in doses not exceeding 1000 mg/month, when it was administered at a stable dose for at least 3 months for interstitial lung disease. However, to reduce the drug related bias, cytometry was performed at least 28 days after the last infusion.

Vasodilators, including calcium channel blockers and angiotensin-converting enzyme (ACE) inhibitors, had to be withdrawn at least 3 days before inclusion (corresponding to more than five times the drug half-life). All patients had white blood cells count within normal range (4 to 10 G/L).

Detailed disease characteristics included damage related from the disease as proposed by Medsger and EUSTAR 2017 scale for disease activity. As a control a group of 20 sex and age matched subjects were recruited.

The study was carried out according to Declaration of Helsinki and study protocol was approved by the local Bioethical Committee at Medical University of Silesia, Poland (approval number KWN/022/KB1/68/16). Patients and controls gave written informed consent prior to any study procedures.

### 2.2. Flow Cytometry

Peripheral blood was taken at rest, in the morning, from the forearm, together with routine analysis in hospitalized patients. Blood samples were collected in tubes containing K_3_ Ethyleno Diamine Tetra Acetic (EDTA) as anticoagulant. For phenotype analysis, samples were stained (direct immunofluorescent technique) with mouse anti-human monoclonal antibodies (MoAb) directed against surface markers. ‘Bulk lysis/wash/stain/wash’ technique, with BD PharmLyse Lysing Solution (15’) and BD Pharmingen Stain Buffer (FBS) (200× *g*, RT, 5’), was used. Leucocytes (0.5–1 × 10^6^ cells per tube) were incubated with mouse anti-human monoclonal antibodies (MoAb): CD14-FITC (clone Leu-M3; 345784), CD24-PE (clone ML5; 555428), CD28-PE (clone Leu-28; 348047), CD3-PerCP-CY5.5 (clone SK-7; 332771), CD34-PerCP-Cy5.5 (clone HPCA-2; 345802), CD16-PE-Cy7 (clone B73.1; 335823), CD19-PE-Cy7 (clone SJ25C1; 341113), CD56-APC (clone NCAM16.2; 341027), CD27-APC (clone L128; 337169), CD38-APC-H7 (clone HB7; 656646), CD8-APC-H7 (clone SK1; 641400), CD4-V450 (clone SK3; 651847), IgG1-V450 (isotypic control, clone X40; 642268), CD45-V500 (clone 2D1; 655873). All antibodies were purchased from Becton Dickinson (Heidelberg, Germany).

The following two antibody combinations were used for the staining of cells:

(1) CD4-V450/CD45-V500/CD14-FITC/CD28-PE/CD3-PerCP-Cy5.5/CD16-PE-Cy7/CD56-APC/CD8-APC-H7
(2) IgG1-V450/CD45-V500/CD14-FITC/CD24-PE/CD34-PerCP-Cy5.5/CD19-PE-Cy7/CD27-APC/CD38-APC-H7

Negative/positive internal controls, unstained cells and negative isotypic controls (IgG1-V450/CD45-V500/IgG1-FITC/IgG1-PE/CD3-PerCP-Cy5.5./CD19-PE-Cy7/IgG1-APC/IgG1-APC-H7 were taken into account to discriminate between the negative and positive fraction.

Stained samples were acquired on a 3-laser, 10-parameter FACSCanto II flow cytometer (Becton Dickinson). The cytometer settings and daily instrument quality control was performed with BD FACSDiva CS &T IVD Beads (656047), BD Multicolor CompBeads kit (BD 644204) and SPHERO^TM^ Rainbow Calibration Particles (Spherotech RCP-30-5A). Blood samples were transferred to the laboratory within 1 h, data acquisition was performed immediately after completion of sample staining. Additionally, automated absolute number of leucocyte (WBC) and leukocyte subsets were obtained on Sysmex XN-10 (Sysmex, Hamburg, Germany). 

**Figure 1:**
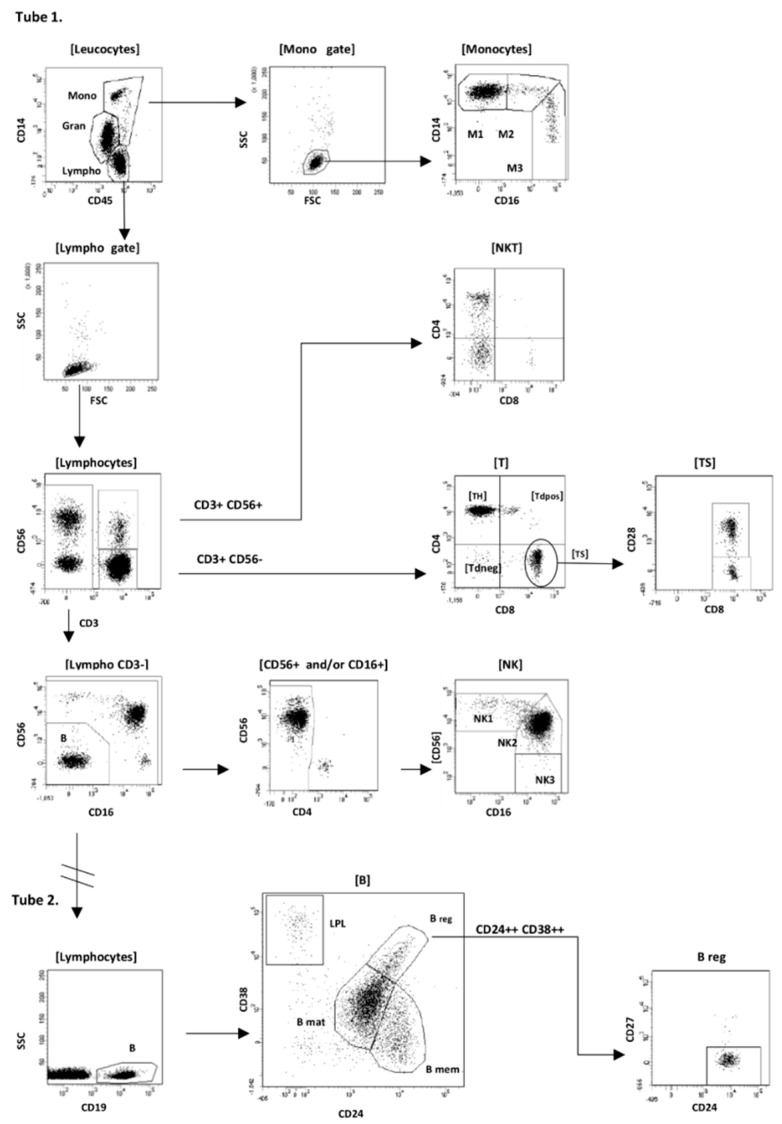
Gating strategy used in the study for immune cells identification. Example of gating strategy for multiparameter flow cytometry analysis of subpopulations of peripheral blood leucocytes of a healthy donor. The first step of analysis was common for both tubes: viable CD45^+^ leucocytes without debris and doublets were displayed on CD45/CD14 dot plot, and gates for monocytes, lymphocytes, and additionally (not analyzed here) granulocytes were gated as shown. Next, the ‘back-gating strategy’ (on FSC/SSC dot plot) was used to purify monocytes (Mono gate) and lymphocytes (Lympho gate) from unwanted cells. Tube 1: Subpopulations of monocytes and NKT, T, and NK cells were examined. For monocytes-classical (M1), intermediate (M2), and non-classical (M3) subpopulations were separated. Lymphocytes were divided into CD3^+^ and CD3^−^ fraction. In the CD3^+^ fraction, CD3^+^CD56^+^ cells (the negative expression of CD16 was not shown) were designated as NKT cells, and CD3^+^CD56^−^ cells were designated as ‘true’ T lymphocytes. On the basis of the co-expression of CD4 and CD8 antigens, four subpopulations of T lymphocytes were established: T helper CD4^+^CD8^−^ (T_H_), T suppressor CD4^−^CD8^+^ (Ts) with CD28^−^ and CD28^+^ cells, T double negative CD4^−^CD8^−^ (T_dneg_), and T double positive CD4+CD8+ (T_dpos_). NK cells were selected from CD3^−^ fraction as CD56^+^ and/or CD16^+^ cells, with CD56^bright^CD16^−^/^+^ subpopulation (sign as NK1 gate on the scheme), CD56^dim^CD16^+^ cells (NK2 gate), and rare CD56^−^CD16^+^ cells (NK3 gate). Additional CD4/CD56 dot plot was previously used to remove residual dendritic cells from NK3 gate. The subpopulation of CD3^−^CD56^−^CD16^−^ cells consisting of B lymphocytes (CD19^+^) was analyzed more precisely in Tube 2. Tube 2: CD19^+^ lymphocytes from Lympho gate were displayed on CD24/CD38 dot plot, and the following main B subpopulations were gated: B regulatory CD38^bright^CD24^bright^CD27^−^(B_reg_), B mature CD38^dim^CD24^dim^ (B_mat_), B memory CD38^−^CD24^bright^ (B_mem_), and lympho-plasmocytes CD38^bright^CD24^−^ (LPL).

Analysis of flow cytometry data was performed using BD FACSDiva software v.7.0 (Becton Dickinson Biosciences, San Jose, CA, USA). Our gating strategy is shown schematically in Figure 1.

Firstly, after the standard rejection of debris, residual erythrocytes and doublets from the analysis, we gated monocytes as CD14^++/+^/CD45^++^/SSC^mid^ FSC^high^ cells, lymphocytes as CD14^−^/CD45^++^/SSC^low^/FSC^low^ cells, and additionally granulocytes as CD14^−/dim^/CD45^+^ cells. In the second step the following subpopulations of monocytes, lymphocytes, and CD34^+^ cells were defined:

(1) B-cell (CD19^+^) with subsets: B regulatory (B_reg_) CD19^+^/CD24^high^/CD38^high^/CD27^−^, B memory (B_mem_) CD19^+^/CD38^−^/CD27^+^/CD24^high^, B mature (B_mat_) CD19^+^/CD24^low^/CD38^low^/CD27^−^, plasmablasts (PL) CD19^low^/CD24^−^/CD38 ^high^/CD27^high^, B naïve (B_naïve_) CD19^+^/CD38^−^/CD27^−^/CD24, B immature (B_imm_) CD19^+^/CD38^low^/CD45 ^high^;
(2) T-cell (CD3^+^/CD56^−^/CD16^−^) with subsets: T helper (T_H_) CD3^+^/CD4^+^/CD8^−^, T cytotoxic/supressor (T_C/S_) CD3^+^/CD4^−^/CD8^+^ (with CD28^+^ and CD28^−^ subpopulations), T double positive (T_dp_) CD3^+^/CD4^+^/CD8^+^, and T double negative (T_dn_) CD3^+^/CD4^−^/CD8^−^;
(3) NKT (CD3^+^/CD56^+^/CD16^−^) cells;
(4) NK cell (CD3^−^/CD56^+^ and/or CD16^+^) with subsets: NK regulatory CD56^high^/CD16^+/^^−^ (NK1), NK cytotoxic CD56^+^/CD16^high^ (NK2), and additionally rare CD56^−^/CD16^+^ cells (NK3);
(5) Monocytes: classical CD14^++^/CD16^−^ (M1), intermediate CD14^++^/CD16^+^ (M2), and non-classical CD14^+^/CD16^++^ (M3).

### 2.3. Statistical Analysis

Statistical analysis was performed using STATISTICA 10.0 PL (Tibco Inc., Palo Alto, CA, USA) and STATA 13.0 (Stata Corp. Lakeway Drive, TX, USA). Statistical significance was set at a *p*-value below 0.05. All tests were two-tailed and any data imputation was done as required. Nominal and ordinal data were expressed as percentages, whilst interval data were expressed as mean value ± standard deviation in the case of a normal distribution or as median with lower quartile and upper quartile in the case of data with skewed or non-normal distribution. Distribution of variables was evaluated by the Shapiro–Wilk test and the quantile-quantile (Q-Q) plot. Homogeneity of variances was assessed by the Fisher–Snedecor test. For comparison of data between two groups the *t*-Student test for independent data was used (in case of normal data distribution) or after the logarithmic transformation (in case of skewed distribution if appropriate). In other cases, the non-parametric *U* Mann–Whitney test was used. The assessment between variables was done based on the Pearson correlation coefficient and with stepwise backward multivariable linear regression models. The Cook–Weisberg test and Cameron & Trivedi’s decomposition test was used to test the residuals for heteroskedasticity as well as the violation of skewness and kurtosis assumptions in linear regression. Multicollinearity was evaluated by calculating the variance inflation factor (VIF), which should not exceed 5. The one way analysis of variance (ANOVA) has been used to compare the treatment groups to the control group and healthy subjects.

## 3. Results

### 3.1. Patients’ Characteristics

A total number of 46 patients with systemic sclerosis were included into the study. The diffuse type of the disease was diagnosed in 36 (78.3%) patients, the limited form in 10 (21.7%) patients. The proportion of dcSSc to lcSSc, although atypical for systemic sclerosis subsets reflects the patient population being covered by our department with a predominance of the more severe dcSSc type who typically require more aggressive treatment and are referred to our department.

The mean age of all patients was 54.5 ± 11.1 years. The mean duration of the disease from first non-Raynauds symptom (attributable to systemic sclerosis) was 6.7 ± 7.8 years in all patients.

The concomitant diseases diagnosed in patients were interstitial lung disease, arterial hypertension, pulmonary arterial hypertension, digital ulcers, arthralgia, and esophagus dysmotility.

Patients underwent immunosuppressive treatment as required on clinical status, that included cyclophosphamide, methotrexate, azathioprine, and mycophenolate mofetil. Twelve patients had no immunosuppressive therapy.

The clinical and demographic characteristics of the SSc patients included are summarized in Table 1. 

### 3.2. Cytometric Characterization of Patients and Controls

We first performed a detailed, multiparameter immune cell profiling by flow cytometry to get a comprehensive overview about immune cell subset composition of SSc patients and compared cell profile to healthy controls. As presented in Figure 1 we identified several lines of immune cell including T, B, NK, NKT, and monocytes, which were further characterized as T helper (Th), T suppressor (Ts), double positive T cells (Tdp CD4^+^CD8^+^), double negative T cells (Tdn CD4^−^CD8^−^). To further characterize the changes in B cell subset composition, we first analyzed cell frequencies and absolute numbers of B cells, that were further characterized as B regulatory (Breg CD19^+^CD24^++^CD38^++^CD27^−^), B memory (CD19^+^CD24^++^CD38^−^CD27^+^), B mature (CD19^+^CD24^low^CD38^low^CD27^+^), and plasmablasts (CD19^+^CD24^−^CD38^+^^−^CD27^++^). NKT and NK cells were identified as having CD3^+^CD56^+^16^−^ and CD3^−^CD56^+^CD16^+^, respectively. 

As compared to healthy subjects, systemic sclerosis patients were characterized by higher frequency of T cells within lymphocytes (mean ± SD; 72.3 ± 13.2 vs. 57.3 ± 9.3). This predominance was also seen when we compared controls to the non-treatment group (*p* < 0.01). However, subanalysis within Th and Ts lines showed that Th and Ts higher frequencies were only seen when we analyzed Th and Ts frequency within total lymphocytes (46.8 ± 13.4 vs. 35.5 ± 8.4, *p* < 0.001 and 22.8 ± 9.7 vs. 17.9 ± 5.4, *p* < 0.05), respectively. Moreover, non-treated SSc patients were characterized by higher Th frequency as compared to healthy controls (*p* < 0.05). Further analysis of T cells showed differences in Tdp (CD4^+^CD8^+^) and Tdn (CD4^−^CD8^−^) cells in the whole SSc group in comparison to heathy controls. In SSc patients, the percentages of Tdp were higher, in parallel to reduction of Tdn. Reductions of Tdn in patients were observed when we analyzed Tdn as a percentage of all T cells and total lymphocytes separately. We failed to observe any changes in regard to CD28 expression between controls and whole SSc patients group. However, when we analyzed the non-treated SSc subgroup, we observed significant reduction of frequency of CD28 T cells in non-treated patients in comparison to controls (54.9 ± 21.4 vs. 73.6 ± 14.8, *p* < 0.01).

In the study, NKT (CD3^+^CD56^+^CD16^−^) and NK (CD3^−^CD56^+^CD16^+^) cells were identified. In the subanalysis NK cells were further identified as: NK regulatory CD56 **^high^**/CD16^+/^^−^ (NK1), NK cytotoxic CD56^+^/CD16^high^ (NK2), and CD56^−^/CD16^+^ cells (NK3). As compared with healthy controls, NK absolute count was lower in SSc patients corresponding to the reduced frequency of NK cells within all lymphocytes. Absolute NKT count was significantly reduced in SSc patients, however we did not observe any changes in frequencies of NKT cells, when we analyzed frequencies of NKT within total lymphocytes (*p* = 0.14) and within T cells separately.

When we analyzed B cell populations, we observed a reduction in absolute B cell count in non- treated patients with SSc as compared to controls, moreover a reduction in the frequency of memory B cells (as a percentage of all CD19) was observed in the whole SSc group in comparison to healthy subjects. In line with this, increased frequency of plasmablasts were seen. Of note, increased frequencies of plasmablasts were seen when we analyzed plasmablasts in the whole SSc group and non-treatment group respectively. In contrast, we observed only a mild reduction of CD19^+^ frequency within total lymphocytes in whole SSc patients compared with controls (*p* = 0.09). Surprisingly, analysis of the non-treated group versus controls gave the opposite results where the frequency of CD19 was higher than in controls, but lower when we compared absolute CD19 counts (Table 2). Analysis of Breg population failed to show any differences between patients and controls. Frequency of Breg, although numerically higher in SSc patients, did not reach the threshold for statistical significance (*p* = 0.21). 

In the next part of the study analysis of monocytes was performed. We identified classical monocytes (CD14^++^CD16^−^), intermediate (CD14^++^CD16^+^) and non-classical (CD14^++^CD16^++^). Among subpopulations of monocytes studied, only the frequency of intermediate monocytes was statistically higher in SSc patients than in healthy subjects. All cytometric results were summarized in Table 2.

### 3.3. Immune Cell Profile in Relation to Disease Activity and Severity

In the last part of the study, we addressed whether changes in immunocompetent cells might have influence on disease activity and severity. As at first approach, we performed an analysis of alteration in population of cells studied. Then we identified the subgroups of patients with typical forms of systemic sclerosis-related damage including digital ulcerations, pitting scars, pulmonary artery hypertension, and arthralgia. In general, patients with active digital ulcerations had higher level of T cells, but lower percentage of mature B cells when compared to SSc patients free of these symptoms. In regression analysis, we additionally showed that patients with digital ulceration have higher frequencies of Breg within all CD19^+^ (β = 6.60; SE(β) = 2.82; *p* < 0.05).

Additionally, we showed that in patients with pitting scars (that represents next step of digital ulceration) only reduction of NKT cell frequencies (as a percentage of all lymphocytes) were observed. 

Pulmonary arterial hypertension represents the most serious complication of the disease, accounting for premature death in this group of patients. The role of B lymphocytes in the pathogenesis of PAH, was recently intensively debated as the B cells are not only precursors of plasma cells producing autoantibodies, but also play a key role in cell-mediated immune regulation. Regression analysis within patients with pulmonary hypertension showed reduced frequencies of Breg within all CD19^+^ (β = –6.45; SE(β) = 2.96; *p* < 0.05). In this subgroup of patients, reduction of frequencies of B memory within all CD19^+^ were also observed.

SSc patients with PAH were also characterized by a higher frequency of intermediate monocytes (percentage within all monocytes) (β = 0.20; SE(β) = 0.08; *p* < 0.05). Contrary to this, we did not find any changes in cell populations studied in patients with interstitial lung disease.

Finally, patients with arthralgia had a higher percentage of plasmablasts and intermediate monocytes (β = 0.13; SE(β) = 0.06; *p* < 0.05), but lower NK and Th cell as compared to subjects who did not have arthralgia (*p* < 0.05 for all cell lines) (Table 3).

To assess the potential correlation of peripheral immune profile with occurrence of clinical disease activity, disease-related damage and disease duration correlation studies were performed. We used separate models of disease activity and disease-related damage. In the study, values on Medsger scale correlated positively with total lymphocytes and negatively with NKT cells. Moreover, when we analyzed CD19 population, we found significant correlation between the frequencies of CD19^+^ and plasmablasts and disease activity measured according to EUSTAR 2017 scale, reverse for CD19 (*R* = −0.32), and positive for plasmablasts (*R* = 0.32), respectively (Table 4).

To get some insight into the role of immune profiles in patients with systemic sclerosis and potential impact of Scl-70 and anti-centromere antibody (ACA) positivity on immune cells components and disease type and activity, a correlation study in patients with anti-Scl-70 and ACA antibody positivity was also performed. In the study, patients who were positive for ACA had lesser extent of skin involvement, lower disease severity (Medsger severity scale) and disease activity (EUSTAR 2017). Moreover ACA positive patients were characterized by a higher number of NK cells (Table 5). We did not show any other relationship between populations of cells studied and autoantibody profile, extent of skin involvement, disease activity, and severity.

### 3.4. Impact of Immunosuppressive Treatment on Immune Cell Profile

This is a real-world clinical study where patients underwent several types of treatment including strong immunosuppressants, therefore we also analyzed cell populations separately in subgroups who were treated with cyclophosphamide and mycophenolate mofetil. Treatment with cyclophosphamide when compared with other forms of treatment contributed to a lower frequency of CD19 cells. Contrary to this, mycophenolate mofetil administration resulted in a significant increase in frequency of plasmablasts within all CD19^+^ cells.

Finally, head to head comparison between cyclophosphamide and mycophenolate was done showing reduction of frequencies of CD19^+^ cells but increment of plasmablasts within CD19^+^ cells in patients treated with cyclophosphamide (Table 6).

To compare the effect of immunosuppressive treatment, we performed analysis in three groups; namely cyclophosphamide (CYC), mycophenolate (MMF), and immunosuppressive treatment free. One-way ANOVA demonstrated that there were statically significant differences between non-treated, CYC, and MMF patients in CD19 frequency within all lymphocytes (*p* < 0.05). Patients treated with CYC have significantly lower CD19 frequencies than non-treated (*p* < 0.05). No differences in CD19 were observed between non-treatment SSc patients and the MMF group (*p* = 0.77). Moreover, significant differences between all three groups were observed regarding frequency of plasmablasts within all CD19 (*p* < 0.001). However, detailed analysis in this cell subpopulation showed only higher frequency of plasmablasts in the CYC group in comparison to MMF (*p* < 0.05). No significant changes were observed in MMF and CYC groups in comparison to treatment free patients (*p* = 0.09 and *p* = 0.08, respectively).

## 4. Discussion

In the current study, we aimed to characterize immunocompetent cells populations in patients with systemic sclerosis in a real world clinical setting using flow cytometry. All patients had an established diagnosis of SSc and were characterized by the long duration of disease. Patients were allowed to take all treatment as required by clinical status. One-third of patients were treatment-free and served as a second control group enabling a direct comparison between controls and treatment free SSc patients. Moreover, we did not exclude patients treated with cyclophosphamide unless its administration was due to concomitant interstitial lung diseases related to SSc. As a result, we received real-word cytometric characteristics of main populations of B, T, NK, and NKT cells and monocytes in patients who were routinely treated for systemic sclerosis. We also tried to find a link between changes in main populations of immunocompetent cells and disease severity and activity.

In the study, we showed that both cyclophosphamide and mycophenolate mofetil had an impact on frequencies of cells studied. To clarify that, we performed a separate analysis of CYC and MMF influence and compared head to head the effect of both immunosuppressants. Specifically, CYC treatment contributed to the significant reduction of B cells and small non-statistical increment in frequencies of NKT cells. These findings are in perfect line with results of a previous study in lupus patients comparing CYC and MMF where CYC treatment resulted with more pronounced reduction of B cells than MMF [16]. The same is true as far as plasmablasts are concerned. In the same study, Fassbinder et al. showed a marked reduction of plasmablasts in the MMF treatment arm in comparision to cyclophospahmide regimen, that is again in the perferct line with the result from our study [16]. It may suggest that in spite of different types of immune response in lupus and systemic slerosis both immunosupressants act in the same way. The reduction of B cells observed in our study may potentially indicate CYC as a preferable drug for beginning the treatment of SSc, to stop initial steps of immune response driven by B cells and continue the treatment with mycophenolate which supress plasmablast formation. 

In our study, patients treated with MMF exhibited higher frequency of CD8^+^CD28^+^ cells within all CD8^+^ lymphocytes, thus such a treatment may contribute to the reduction of a potentially aggressive type of CD8^+^CD28^−^ lymphocytes. This nummerically marked reduction has been observed in the study. However, it did not reach treashold for statistical signifficance. This observation may be potentially important in the light of established role of CD28 negative T cells in the pathogenesis of systemic sclerosis. Moreover, it may provide the link between the treatment with MMF and the reduction of aggressive subpopulation of CD8^+^CD28^−^ cells, thus explaining the beneficial role of MMF treatment in SSc.

In the study, we observed several significant changes within T cell populations. Patients with SSc exhibited a significant increment in T cell frequency with parallel increment within the Th and Tc compartment. This is opposite to previous studies indicating a reduction of T cells line or a lack of differences in SSc patients [4,12,17]. It is not clear whether these discrepancies came from treatment regimen or are due to patients’ characteristics. It is important to underline that an increase of T cells in SSc patients was seen when the whole SSc goup and non-treatment group were compared to controls seperately. This enables us to rule out drug influence on absolute T cell count and frequency. It is worth noting that, in the study of Lopez-Cacho et al. [17], patients were allowed to take immunosupressants, since the second study examinated only drug-free patients [4]. The same discerpancies come from the study of Almeida et al. [12]. In this study however the majority of patients presented with the limited form of the disease and no immunosupressive treatment was given, that may at least theoretically explain the observed differences. Of note however, is the fact that the majority of our patients were treated with cyclophosphamide and cytometric assessment was performed before the next sheduled infusion. Quite recently it has also been proposed that cyclophosphamide treatment contributes to effector T cell expansion during a so-called ‘rebound’ phase due to homeostatic proliferation following lymphodepletion [18]. However, the same increases in T and Th cells were seen in the group of non-treated SSc patients. Therefore, it is plausible that treatment with cyclophosphamide may only consolidate changes in T cell count and frequencies initially evoked by the disease. The results from our study pointed to the role of T cells in disease activity and damage as T and Th frequencies correlated with Medsger damage scale and Th frequency additionally with EUSTAR 2017 activity index. As the pathogenesis of systemic sclerosis is still a matter of debate, these findings suggest the direct influence of T cells on disease activity in SSc. Moreover, T cell predominance observed amongst patients with systemic sclerosis is not ameliorated with immunosuppressive treatment with either cyclophosphamide or mycophenolate. This indicates that CYC and MMF’s mode of action in systemic sclerosis is not mediated by influence on T cells.

When we determined CD28 expression in the whole SSc patient group, we found that SSc patients exhibited a trend toward reduced expression of CD28 molecules when compared to healthy controls (*p* = 0.08). This is in perfect line with previously published study reporting a higher frequency of CD28 negative T cells and their contribution to fibrotic processes [11,19]. Contrary to the published studies however, we failed to show any significant relationship between the count, frequencies of CD8^+^CD28^−^ T cells, and disease duration, subtype, and activity.

Within the T cell populations, we further assessed the expression of CD4 and CD8 molecules that enabled us to characterize CD4CD8 double positive (Tdp) and double negative (Tdn) T cells. The results from our study confirmed the previously reported observation of increased frequency of CD4CD8 Tdp cells in systemic sclerosis [20]. This finding is of special interest in light of the role Tdp cells play in fibrotic processes in systemic sclerosis. Striking discrepancies exist in the literature regarding the count and frequency of CD4 and CD8 cells. Almeida et al. reviewed data on CD4 expression from 10 studies [12]. Half of them reported reduced frequencies/number of CD8 cells while the remaining ones showed normal values, thus our findings on elevation of CD8 cells is reported for the first time. The same is true as far as CD4 is concerned. CD4 is generally reported as reduced with the exception of only one study that showed similar to our increment in CD4 frequency [21].

Finally, we analyzed NK and NKT cell frequencies and absolute numbers and correlated them with signs of disease exacerbation. In agreement with previous data, we observed a reduction in absolute count and/or percentage of NK and NKT cells in patients with SSc [12,22,23]. Of note is the fact that NK and NKT cells were reduced in patients with arthralgia and pitting scars respectively. Moreover, NKT but not NK counts correlated negatively with disease damage (Medsger scale) and disease duration. This indicates the role of NKT cells in the early phase of disease that is gradually reduced with disease duration [12]. Contrary to this, total lymphocytes—and to a lesser extent Th cell frequencies—may be linked with disease-related damage (Medsger scale).

Intriguingly, for B cells, our cytometric analysis revealed only few changes. However those may be of special importance as CD19^+^ correlated negatively with disease activity, contrary to plasmablasts for which positive correlation with disease activity has been observed. In line with this, two significant changes were observed within B cells compartment, specifically the reduction in frequencies of memory B cells and elevation of plasmablasts within all CD19 cells. It is not a surprising finding in SSc, where the role of B cells is intensively debated [24,25]. Decrease of memory B cells has been already reported in the literature [20]. While reduction of B memory cells is a common finding in SSc patients, results are sometimes conflicting regarding plasmablasts [5,26]. To best of our knowledge, elevation of plasmablasts observed in this study is reported for the first time. Quite recently, Dumoitier et al. reported similar counts of main B cells subsets and reduction of B memory cells between SSc patients and healthy controls [27]. This is in striking dissimilarity with the results from our study. There are several reasons for these discrepancies. Firstly, patients enrolled to our study underwent routine treatment while the other studies investigated mainly drug-free populations. Secondly, plasmablasts are precursors of plasma cells and are rapidly deposited in bone marrow and in the skin of SSc patients, where they rapidly transform to mature plasma cells [28]. Several cytokines, chemokines, and ligands promote homing of plasmablasts to the sites of inflammation. The expression of these homing factors may be reduced during the immunosuppression. Thus, we may speculate that homing of plasmablasts is reduced in the patients on immunosuppression resulting in retention of plasmablasts in peripheral blood. 

The study suggested the potential role of T and B cell in pathogenesis and clinical course of systemic sclerosis.

Of note is the fact that in this study, T cells frequency changes mainly reflect the severity of disease and correlated with Medsger scale and disease duration. Negative relationships between double positive T cell and disease duration indicate the role of this subset of T cells in the early stages of disease whereas for CD19 and plasmablasts, significant correlation with disease activity (EUSTAR 2017) has been observed. This finding may provide new insight into SSc pathogenesis and support the pathogenic role of both T and B cells acting at various stages of the disease-autoantigen recognition (T cells) and the autoantibodies formation in later stages (B cells and plasmablasts). When we analyzed internal organ involvement according to the Medsger scale in relation to changing of immunocompetent cell counts and frequencies, to our surprise, we did not observe any changes in patients with SSc-associated interstitial lung disease. The reason for this is currently unknown, especially in the light of very strong immunosuppression (cyclophosphamide and mycophenolate mofetil) given to this group of patients. Finally, we observed reduction of frequencies of Breg and B memory cells in subgroup with pulmonary hypertension. Although the role of immune response in the initiation and progression of pulmonary artery hypertension is the subject of debate, the role of B cells in PAH in general and PAH in systemic sclerosis in particular has not been studied yet [29]. In this light, the reduction of subtypes of B cells is a new finding indicating involvement of B cells in the development of PAH, although the role and mechanism of this involvement should be elucidated.

In the study, we showed higher populations of CD14^high^ CD16^+^ monocytes in SSc patients than in healthy controls. This finding is in line with the previous study of Highashi-Kuwata et al. which reported higher expression of CD14 molecules in monocytes of patients with SSc [30].

Moreover, in our study CD14^high^CD16^+^ monocytes correlated with presence of PAH. This may potentially indicate the role of CD14^high^ monocytes in the development of pulmonary arterial hypertension in SSc patients. The role of the other monocyte subpopulation, CD14-positive monocytes, has been addressed by Trombetta et al. who showed an association between CD14 positive mixed M1/M2 cell subsets (CD14^+^CD206^+^CD163^+^CD204^+^TLR4^+^CD80^+^CD86^+^) and development of SSc-PAH [31]. As Trombetta et al. studies addressed the other subtypes of monocytes it is plausible that both subtypes of monocytes may play the role in the development of PAH in SSc patients. We may only speculate that this distinct subset of monocytes may reflect the initial stage of monocyte polarization and subsequent role in lung involvement as recently proposed [13]. 

Several conclusions come from this study. Firstly, patients with SSc are characterized by higher T cell counts. Both CYC and MMF showed no efficacy in reduction of this T cell predominance. Secondly, in SSc patients a reduction of CD28 expression is seen that potentially gives way to predominance of autoimmune active CD28 negative cells. This trend however may be reduced by the treatment, both with CYC and MMF. Finally, only two main changes are seen in B cell populations. In general, patients with SSc have lower counts of CD19, but higher plasmablasts frequencies that may be reduced with MMF treatment.

In the present study, we attempted to show changes in main immunocompetent cells subsets in patients with systemic sclerosis who underwent routine treatment. Systemic immunosuppression has a moderate impact on populations of cells studied. However, we showed different modes of action of two main immunosuppressive drugs—MMF and CYC—in SSc patients with more pronounced influence of CYC on B cells contrary to MMF which acts mainly on CD28 and plasmablasts. This may indicate CYC as a drug that should be used in more aggressive form of disease, followed by MMF for remission maintenance.

## 5. Limitations of the Study

Some limitations of the study should be addressed. Firstly, the study comprised 46 patients with systemic sclerosis, which is a large cohort in relation to the size of the population with SSc. In terms of statistical analyses, this group is still small and it may have a negative impact on final calculations resulting in statistical bias. Secondly, for the study, we enrolled unselected patients who were under routine treatment with only 12 treatment-free subjects. On one side, it was an advantage of the study that gave an insight into changes of immunocompetent cells in a real unselected population, but treatment alone—although detailed and separately analyzed—obviously exerted a strong impact on the final result. Therefore, we cannot exclude that several changes may not be directly attributed to the disease, but reflect at least partially drug induced changes. Comparison between controls and a small treatment-free patient group is obviously not free of statistical bias. Finally, some of our results are in direct opposition to the results already published; therefore, our data should be verified—perhaps in a larger cohort of patients.

## Figures and Tables

**Table 1:** Demographic characteristics, clinical severity, and disease damage in patients with SSc.

Parameter						Mean ± SD or *n* (%)				
Patients (*n* = 46)										Controls (*n* = 20)
Gender (Female/Male)						31/15				11/9
Age (years)						54.5 ± 11.1				53.9 ± 12.5
Disease duration since first non-Raynaud’s symptom (years)						6.7 ± 7.8				
Disease duration (since formal diagnosis) (years)						4.5 ± 2.3				
Age at the time of disease diagnosis (years)						48.7 ± 12.0				
Disease type										
Diffuse type (dcSSc), *n* (%)						36 (78.26%)				
Limited type (lcSSc), *n* (%)						10 (21.74%)				
mRSS (points)						11.1 ± 9.5				
EUSTAR 2017 (points)						2.1 ± 2.1				
Autoantibody presence, *n* (%)										
ANA						46 (100)				
Topoisomerase I (SCL-70)						29 (63.04)				
CENP-B						10 (21.74)				
SSA. SSB. Ro52						8 (17.39)				
Clinical characteristics										
Interstitial lung disease SSc-ILD, *n* (%)						27 (58.69)				
Esophagus dysmotility, *n* (%)						24 (52.17)				
Arthralgia, *n* (%)						19 (41.30)				
Pitting scars, *n* (%)						12 (26.08)				
Digital ulceration, *n* (%)						9 (19.56)				
Arrhythmia, *n* (%)						9 (19.56)				
Pulmonary arterial hypertension, PAH, *n* (%)						8 (17.39)				
LVEF < 50%						4 (8.69)				
Digital necrosis, *n* (%)						3 (6.52)				
Treatment										
Mycophenolate mofetil (MMF), *n* (%)						19/46 (41.3)				
Cyclophosphamide (CYC), *n* (%)						11/46 (23.9)				
Azathioprine (AZA), *n* (%)						2/46 (4.3)				
Metotrexate (MTX), *n* (%)						2/46 (4.3)				
Disease severity according to Medsger scale										
	General health	Peripherial vascular	Skin	Joints/tendons	Muscles	GI	Lungs	Heart	Kindey	
No changes, *n* (%)	37 (80.4)	8 (17.4)	5 (10.9)	11 (23.9)	37 (80.4)	23 (50.0)	15 (32.6)	38 (82.6)	45 (97.8)	
Mild, *n* (%)	6 (13.0)	22 (47.8)	27 (58.7)	24 (52.2)	7 (15.2)	23 (50.0)	23 (50.0)	2 (4.3)	0 (0.0)	
Moderate, *n* (%)	2 (4.3)	10 (21.7)	12 (26.1)	9 (19.6)	2 (4.3)	0 (0.0)	4 (8.6)	1 (2.2)	1 (2.2)	
Severe, *n* (%)	1 (2.2)	4 (8.6)	2 (4.3)	2 (4.3)	0 (0.0)	0 (0.0)	2 (4.3)	5 (10.9)	0 (0.0)	
End-stage, *n* (%)	0 (0.0)	2 (4.3)	0 (0.0)	0 (0.0)	0 (0.0)	0 (0.0)	2 (4.3)	0 (0.0)	0 (0.0)	

mRSS, modified Rodnan Skin Score; ANA, antinuclear antibody; CENP-B, anti-centromere B antibody; PAH, pulmonary arterial hypertension; LVEF, left ventricle ejection fraction; SSA, SSB, Ro52, antibody anti Ro, La and Ro53 antigens respectively.

**Table 2:** Main immunocompetent cells in patients with systemic sclerosis and healthy subjects.

No.	Type of Cells	Systemic Sclerosis (*n* = 46)	Controls (*n* = 20)	*p*
1	lymphocytes T (%)	72.3 ± 13.2	57.3 ± 9.3	<0.001
2	Th (%) within all lymphocytes	46.8 ± 13.4	35.5 ± 8.4	<0.001
3	Th (%) within T lymphocytes	64.4 ± 13.6	61.9 ± 9.8	0.46
4	Ts (%) within all lymphocytes	22.8 ± 9.7	17.9 ± 5.4	<0.05
5	Ts (%) within T lymphocytes	31.7 ± 12.6	31.5 ± 8.8	0.94
6	Ts CD28^+^ (%Ts)	64.9 ± 20.4	73.6 ± 14.8	0.08
7	Ts CD28^−^ (%Ts)	35.1 ± 20.4	26.4 ± 14.8	0.08
8	Tdp (%) within all lymphocytes	0.57 (0.38–0.94)	0.45 (0.33–0.61)	<0.05
9	Tdp (%) within lymphocytes T	0.84 (0.57–1.50)	0.90 (0.49–0.98)	0.37
10	Tdn (%) within all lymphocytes	1.47 (0.81–1.98)	2.24 (1.68–2.90)	<0.01
11	Tdn (%) within lymphocytes T	2.07 (1.03–2.78)	3.87 (2.98–4.80)	<0.001
12	NK (%) within all lymphocytes	7.48 (4.70–9.83)	21.30 (16.77–29.32)	<0.001
13	NK (count × 10^3^/µL)	0.11 (0.08–0.15)	0.47 (0.36–0.75)	<0.001
14	NKT (%) within all lymphocytes	3.57 (2.25–6.90)	4.49 (2.81–9.34)	0.14
15	NKT (count × 10^3^/µL)	0.09 ± 0.08	0.17 ± 0.14	<0.05
16	Th (count × 10^3^/µL)	0.81 ± 0.43	0.88 ± 0.32	0.41
17	Ts (count × 10^3^/µL)	0.33 (0.19–0.46)	0.44 (0.33–0.53)	0.06
18	Th/Ts	2.26 (1.55–3.41)	1.73 (1.57–2.63)	0.72
19	14^++^16^−^ classic (% MONO)	85.0 (76.3–88.3)	83.8 (80.3–87.1)	0.97
20	14^++^16^+^ intermediate (% MONO)	5.9 (4.2–7.5)	4.1 (2.9–5.3)	<0.01
21	14^+^16^++^ non-classical (% MONO)	9.0 (5.8–15.1)	10.3 (9.1–14.0)	0.12
22	% CD19^+^ within all lymphocytes	9.5 (4.2–16.1)	10.5 (8.3–12.8)	0.09
23	% B reg within all CD19^+^	6.8 (2.1–9.3)	4.6 (4.2–6.4)	0.21
24	% B mem within all CD19^+^	13.7 (7.5–20)	17.7 (15–25.7)	<0.01
25	% B mature within all CD19^+^	60.0 (53.6–76.0)	66.9 (60.2–73.2)	0.92
26	%B plasmablasts within all CD19^+^	0.44 (0.11–1.65)	0.27 (0.07–0.50)	<0.05
Non-treatment SSc group vs. healthy controls				
	**Healthy controls (*n* = 20)**	**Non-treatment SSc patients (*n* = 12)**	***p***	
T cell (% within all lymphocytes)	57.3 ± 9.3	71.5 ± 15.0	<0.01	
Th CD4^+^ (% within all lymphocytes)	35.5 ± 8.4	44.2 ± 12.3	<0.05	
Ts CD28^+^ (% within Ts)	73.6 ± 14.8	54.9 ± 21.4	<0.01	
CD19^+^ (absolute count)	0.224 + 0.171	0.087 ± 0.117	<0.05	
CD19^+^(% within all lymphocytes)	10.5 (8.3–12.8)	14.1 (4.5–20.3)	<0.01	
Plasmablasts (% within all CD19^+^)	0.27 (0.07–0.50)	0.50 (0.14–2.50)	<0.001	

Data presented as mean ± SD or median and lower and upper quartile where appropriate. Th, T helper cells; Ts, T suppressor cells; Tdp, double positive T cells (CD4^+^CD8^+^); Tdn, double negative T cells (CD4^−^CD8^−^); NK, natural killer cells; NKT, natural killer T cells; MONO, monocytes; Breg, B regulatory cells.

**Table 3:** Changes in count/frequencies of immunocompetent cells in relation to organ involvement in patients with systemic sclerosis.

No.	Cells	Digital Ulcerations (*n* = 9)	Digital Ulceration Free (*n* = 36)	*p*
1	Limf T (%)	79.4 ± 13.9	70.6 ± 6.1	<0.01
2	% B mature within all CD19	52.9 (36.2–65.5)	67.4 (59.4–76.8)	<0.01
		**Pulmonary hypertension present (*n* = 8)**	**Pulmonary hypertension absent (*n* = 38)**	***p***
3	% B mem within all CD19	20.0 (12.1–32.5)	21.1 (16.9–25.5)	0.07
	Monocyte classical CD14^high^CD16^−^	80.1 ± 9.5	81.6 ± 9.9	0.70
	Monocyte intermediate CD14^high^CD16^+^	8.1 (7.3–12.0)	5.4 (4.0–7.2)	<0.05
	Monocyte non-classical CD14^+^CD16^high^	7.8 (95.2–13.0)	9.8 (5.8–15.1)	0.62
		**Arthralgia present (*n* = 19)**	**Arthralgia absent (*n* = 27)**	***p***
4	NK (count × 10^3^/µL)	0.09 (0.06–0.14)	0.14 (0.08–0.17)	<0.05
5	Th (count × 10^3^/µL)	0.66 ± 0.29	0.90 ± 0.48	<0.05
6	%B plasmablasts CD19	0.75 (0.15–2.64)	0.36 (0.07–1.01)	0.07
		**Pitting scars present (*n* = 1** **2)**	**Pitting scars absent (*n* = 34)**	***p***
7	NKT (%) within all lymphocytes	2.34 (0.73–4.83)	4.11 (2.65–7.35)	<0.05

Data presented as mean ± SD or median and lower and upper quartile where appropriate. NK, natural killer cells; NKT, natural killer T cells.

**Table 4:** Changes in immunocompetent cells in patients with systemic sclerosis in correlation to the disease duration, activity, and severity.

No.	Cells	Medsger Scale	EUSTAR 2017	Log_10_ (Disease Duration)
1	Lymphocytes T (%)	*R* = 0.32; *p* < 0.05		
2	Th (%) within all lymphocytes	*R* = 0.27; *p* = 0.07	*R* = 0.28; *p* = 0.06	
3	log_10_ Tdp (%) within all lymphocytes			*R* = −0.30; *p* < 0.05
4	log_10_ Tdp (%) within lymphocytes T			*R* = −0.31; *p* < 0.05
5	NKT (count × 10^3^/µL)	*R* = −0.32; *p* < 0.05		*R* = −0.36; *p* < 0.05
6	Th (count × 10^3^/µL)			*R* = −0.27; *p* = 0.07
7	log_10_ % CD19^+^ within all lymphocytes		*R* = −0.35; *p* < 0.05	
8	log_10_ % plasmablasts within all CD19		*R* = 0.32; *p* < 0.05	

Th, T helper cells; Tdp, double positive T cells (CD4^+^CD8^+^); Tdn, double negative T cells (CD4^−^CD8^−^); NKT, natural killer T cells; CD19^+^, CD19 positive B cells.

**Table 5:** Relation between anti-centromere antibody (ACA) positivity and NK cells, disease severity, and activity in patients with systemic sclerosis.

	ACA Positive	ACA Negative	*p*
NK (%) within all lymphocytes	8.36 (7.02–13.22)	7.00 (4.09–9.39)	0.076
NK (count × 10^3^/µL)	0.14 (0.116–0.228)	0.09 (0.064–0.146)	<0.05
Skin involvement Rodnan scale	3 (0–6.0)	11 (6.0–18.0)	<0.05
Disease severity Medsger scale	3.5 (1.0–5.0)	6.5 (4.0–9.0)	<0.01
Disease activity EUSTAR 2017	0 (0–1.5)	1.9 (1.0–4.0)	<0.05

Data presented as median and lower and upper quartile. NK, natural killer cells.

**Table 6:** Impact of treatment with cyclophosphamide and mycophenolate mofetil on immune cells in patients with systemic sclerosis.

**Cyclophosphamide Treatment vs. Non-Cyclophosphamide Treatment**			
	**Non-CYC Treatment (*n* = 35)**	**Cyclophosphamide Treatment (*n* = 11)**	***p***
T cells (count × 10^3^/µL)	1.22 (0.79–1.51)	0.88 (0.51–1.38)	0.13
NKT (%within all lymphocytes)	3.29 (2.25–5.57)	7.11 (2.38–14.14)	0.068
CD19^+^ (% within all lymphocytes)	13.36 (4.20–18.62)	4.95 (1.35–7.51)	<0.001
**Cyclophosphamide Treatment vs. Mycophenolate Mofetil Treatment**			
	**Mycophenolate Mofetil Treatment (*n* = 19)**	**Cyclophosphamide Treatment (*n* = 11)**	***p***
Treatment duration (months)	9.1 (6.5–11.0)	3.5 (2.5–7.5)	0.52
T cells (count × 10^3^/µL)	1.24 (0.79–1.51)	0.88 (0.51–1.38)	0.17
CD19^+^ (% within all lymphocytes)	11.55 (4.20–18.87)	4.95 (1.35–7.51)	<0.01
Plasmablasts (% within all CD19^+^)	0.134 (0.024–0.59)	2.187 (0.752–3.33)	<0.01
**Mycophenolate Mofetil Treatment vs. Non-Mycophenolate Mofetil Treatment**			
	**Mycophenolate Mofetil Treatment (*n* = 19)**	**Non-Mycophenolate Mofetil Treatment (*n* = 27)**	***p***
T cells (count × 10^3^/µL)	1.24 (0.79–1.51)	1.14 (0.76–1.39)	0.49
Ts CD28^+^ (% within Ts)	71.55 ± 16.46	60.15 ± 21.84	0.061
Plasmablasts (% within all CD19^+^)	0.134 (0.024–0.59)	0.905 (0.185–2.75)	<0.01

Data presented as mean ± SD, or median and interquartile range, where appropriate.
